# Validation of self - confidence scale for clean urinary intermittent self - catheterization for patients and health - caregivers

**DOI:** 10.1590/S1677-5538.IBJU.2015.0468

**Published:** 2017

**Authors:** Cintia Fernandes Baccarin Biaziolo, Alessandra Mazzo, José Carlos Amado Martins, Beatriz Maria Jorge, Rui Carlos Negrão Batista, Silvio Tucci

**Affiliations:** 1 Programa de Pós-Graduação em Mestrado Profissional de Tecnologia e Inovação em Enfermagem da Escola de Enfermagem de Ribeirão Preto da Universidade de São Paulo - USP - Ribeirão Preto,SP, Brasil;; 2 Departamento de Enfermagem Geral e Especializada da Escola de Enfermagem de Ribeirão Preto, Universidade de São Paulo - USP - Ribeirão Preto, SP, Brasil;; 3 Departamento de Enfermagem Cirúrgica da Escola Superior de Enfermagem de Coimbra, Portugal;; 4 Departamento de Enfermagem Fundamental da Escola de Enfermagem de Ribeirão Preto da Universidade de São Paulo - USP - Ribeirão Preto (SP), Brasil;; 5 Escola Superior de Enfermagem de Coimbra, Portugal;; 6 Divisão de Urologia, Faculdade de Medicina de Ribeirão Preto da Universidade de São Paulo- USP - Ribeirão Preto,SP, Brasil;; 7 Departamento de Cirurgia e Anatomia da Faculdade de Medicina de Ribeirão Preto da Universidade de São Paulo- USP - Ribeirão Preto (SP), Brasil

**Keywords:** Nursing, Intermittent Urethral Catheterization, Gestalt Therapy

## Abstract

**Objective:**

To validate a measurement instrument for clean intermittent self-catheterization for patients and health-caregivers.

**Material and Methods:**

Methodological study of instrument validation performed at a Rehabilitation Center in a University hospital for patients submitted to clean intermittent self-catheterization and their health-caregivers. Following ethical criteria, data were collected during interview with nurse staff using a Likert question form containing 16 items with 5 points each: “no confidence”=1, “little confidence”=2, “confident”=3, “very confident”=4 and “completely confident”=5. Questionnaire called “Self-Confident Scale for Clean Intermittent Self-catheterization” (SCSCISC) was constructed based on literature and previously validated (appearance and content).

**Results:**

The instrument was validated by 122 patients and 119 health-caregivers, in a proportion of 15:1. It was observed a good linear association and sample adequacy KMO 0.931 and X2=2881.63, p<0.001. Anti-image matrix showed high values at diagonal suggesting inclusion of all factors. Screen plot analysis showed a suggestion of items maintenance in a single set. It was observed high correlation of all items with the total, alpha-Cronbach 0.944. The same results were obtained in subsamples of patients and health-caregivers.

**Conclusion:**

The instrument showed good psychometric adequacy corroborating its use for evaluation of self-confidence during clean intermittent self-catheterization.

## INTRODUCTION

Some neurologic diseases affect micturition, including elimination and storage capacity, or complete emptying of bladder, resulting in neurogenic bladder. Usually it is caused by neurological disturbances due to function, obstruction or inability to voluntary control of micturition (1).

Medical diagnosis of neurogenic bladder is established based on clinical exam, laboratory and image exams (X-rays, ultrasound), urodynamic evaluation and clinical behavior of patient (2). Its prognosis is related to early diagnosis and adequate treatment. The objective of treatment is to preserve urinary tract, preventing infections and other complications (3) as well as to reintegrate patients to their daily activities.

Among several treatment alternatives, clean intermittent self-catheterization has been used since 1947. However, only from 1972 on, the technique was widely accepted, using clean and no sterile technique, without prejudice to patients (4).

However, the procedure harms daily activities of patients and their relatives, is bothersome and mostly is continuous, and must be performed several times a day. During clean intermittent self-catheterization (CISC), it is usual to observe difficulty to obtain adequate material and familial support that may cause depression and withdrawal of daily activities related to health care (5). Success of clean intermittent self-catheterization technique improves self-esteem, return to daily micturition routine and also to daily activities (6). In that context, the role of rehabilitation nurses and the use of adequate strategies to warrant compliance of patient and family members are very important. Some authors point out that patient’s confidence is related to education and follow-up strategies (7). Also, self-assurance to perform the procedure stimulates a better health care (8, 9).

Self-confidence is always related to behavior or tasks. Frequently, it is related to repetition and perception of weaknesses and individual potentialities (8). It refers to individual judgement of skills to organize and execute action plans required to achieve behavioral patterns (8).

In daily routine of a rehabilitation center of a University Hospital, we have been seen patients that use clean intermittent self-catheterization and searched instruments that support continuity of treatment, monitoring patient’s self-confidence. Since those instruments are unavailable, we decided to validate a questionnaire. Based on literature, we constructed a question form (8, 10-13) and decided to validate it in relation to form and content in order to evaluate self-confidence of patients and health-caregivers. The instrument was called “Self-Confidence Scale for Clean Intermittent Self-Catheterization” (SCSCISC). It is a Likert question form of 16 items and 5 points per item: “not confident”=1, “little confident”=2, “confident”=3, “very confident”=4, and “completely confident”=5.

The questionnaire was validated in relation to appearance and content in two steps. At first step, a group of 7 specialists, including urologic nurses and physicians of the Rehabilitation Center validated the question form, and in the second step, it was validated by a group of 9 patients and health-caregivers that used clean intermittent self-catheterization (14-15). In the present study, the objective was to validate SCSCISC as an instrument for measurement of self-confidence of patients and health-caregivers.

## MATERIALS AND METHODS

Methodological study of instrument validation performed at a rehabilitation center of a University Hospital of Sao Paulo State, Brazil.

### Sample

The study was developed in patients and their health-caregivers with neurologic bladder, that used intermittent self-catheterization of urinary bladder, and that participate in a rehabilitation program. The sample included 241 subjects (122 patients and 119 health-caregivers).

### Self-Confidence Scale for Clean Intermittent Urinary Self-Catheterization (SCSCISC)

This is a Likert-type question form including 16 items graded with five points: “not confident”=1, “little confident”=2, “confident”=3, “very confident”=4, and “completely confident”=5 (14, 15).

### Data collection

Data collection for validation of the scale was performed by the authors during a nurse consulting routinely performed at the rehabilitation program.

Patients and health-caregivers were interviewed and answered a socio-economic question form and SCSCISC.

### Data analysis

Data were codified and doubly applied in Excel spreadsheets and posteriorly exported and analyzed by SPSS program (Statistical Package for Social Science), version 22.0. S.

In order to determine validity and reliability of scale, it was used descriptive statistics, measuring central tendency and dispersion (media, mode, medium, percent, variance, standard deviation) in order to verify sample adequacy (16) and statistical inference (factorial analysis and estimate of intern consistency). In order to evaluate obtained results, p<0.05 was assumed as statistically significant.

### Ethical Aspects

This study was approved by the Ethical Committee in Research of the Nurse School of Ribeirao Preto (number 146/2012). According to resolution # CNS 466/2012, patients and health-caregivers were invited to participate on the study and signed a free consent form. Anonymity and liberty to quit were maintained throughout the study.

## RESULTS

During the period of the study 241 subjects were interviewed, including 122 (50.2%) patients and 119 health-caregivers (49.4%). 84 (70.6%) health-caregivers were related to patient (mother, father, brother, wife).

Among patients, 78 (64.0%) were male and among health-caregivers 85 (71.4%) were female. In relation to age, most patients were 11 to 20 years old and health-caregivers 31 to 40 years old.

In relation to urinary catheterization, almost all patients (106, 86.9%) and health-caregivers (106, 89.0%) were trained at the hospital of the study. In relation to frequency, most performed catheterization four times a day.

SCSCISC showed good suitability of the database, in a proportion of 15:1, in relation to case number and respective quantity of variables.

There was a good linear correlation among variables (100% of correlation superior to 0.30).

Kaiser Meyer Olking suitability test of sample showed proper adequacy of sample for analysis (16), with a value of 0.931. Using Barlet sphericity test it was obtained statistically significant values with X^2^=2881.63 p<0.001, indicating the existence of relation of the variables.

Anti-image matrix corroborates to sample adequacy of each variable to be used in factorial analysis, with elevated values at diagonal (0.896 “what to do when there is blood in the urine” to 0.964 “how to withdraw the catheter”), suggesting inclusion of all variables for factorial analysis.

In order to obtain the factors of SCSCISC, it was performed a factorial analysis of the main components of the 16 items of scale using the main component method and orthogonal varimax rotation.

Following analysis and Screen-plot observation it was possible to identify the suggestion of items maintenance in one single set. Considering the data, the sample size, the factorial analysis, the Screen-plot convergence and that scale division explained 56.5% of variance, it was maintained, at final analysis, only one set of factors at the scale.

As explained in Table-1, it was checked the variance proportion of each variable, explained by the extracted components (communalities) and factorial burden of each item. Due to the size of the sample, it was decided to maintain the items with factorial burden superior to 0.40 (17).

**Table 1:** Matrix of correlation of items of Varimax rotated factors with Kaiser normalization for a single factor (N=241).

Items	Single Factor 1
I feel I am capable of	
1 - Perform urinary catheterization	0.572
2 - Choose the best time to perform the procedure	0.556
3 - Choose the correct material to perform the material	0.648
4 - Wash my hands	0.719
5 - Perform genital hygiene	0.744
6 - Open the material	0.723
7 - Choose to use or not the lubricant	0.437
8 - Insert the catheter	0.624
9 - Verify how much catheter must be inserted	0.666
10 - Decide how much time to keep urine dripping	0.710
11 - How to withdraw the catheter	0.746
12 - To measure the collected urine	0.654
13 - What to do when there is blood in the urine	0.624
14 - What to do when there is no urine	0.631
15 - How to discard urine	0.609
16 - How to write down the obtained quantity of urine	0.645

In relation to analysis of items set that compose SCSCISC and their relation to data using the Cronbach alpha test (Table-2) testing the proposed items and their correlation, it was obtained a high correlation of all items with the whole scale, resulting in a high alpha value (0.944). All items contributed to the good alpha value; the suppression of any item would harm the scale.

**Table 2:** Homogeneity of Items and Cronbach Internal Consistency Coefficients of SCSCISC scale in totality (N=241).

Items	Medium	Standard Deviation	Correlation with total (corrected)	Alpha if item is suppressed
1	3.90	1.062	0.675	0.941
2	3.63	1.084	0.684	0.941
3	3.85	1.038	0.738	0.940
4	4.24	975	0.685	0.941
5	4.18	982	0.718	0.940
6	4.12	1.018	0.759	0.940
7	3.71	1.258	0.568	0.944
8	3.99	1.074	0.716	0.940
9	3.78	1.095	0.775	0.939
10	3.78	1.152	0.808	0.938
11	3.91	1.033	0.819	0.938
12	3.72	1.145	0.736	0.940
13	2.73	1.431	0.562	0.945
14	3.00	1.386	0.609	0.944
15	3.95	1.069	0.737	0.940
16	3.75	1.233	0.712	0.940

Due to impossibility to use SCSCISC in a completely new sample, the sample was divided in two sub-samples (A and B) obtained by randomization provided by SPSS^®^. At the analysis of subsamples the tests of original sample were replicated and it was observed similar results in relation to reliability of the scale (Cronbach alpha of subsample A 0.942 and 0.931 of subsample B), good correlation and maintenance of scale with a single set of factors.

Also, the sample was divided in two categories (PATIENTS and HEALTH-CAREGIVERS). In these subsamples the same testes were again replicated and it were found similar results related to reliability of the scale (Cronbach alpha PATIENT 0.947 and HEALTH CAREGIVER 0.940), good correlation and maintenance of scale with only one set of factors.

The results are presented in Table-3.

**Table 3:** Descriptive statistics of every dimension of the scale.

Items	N	Minimum	Maximum	Medium	Standard deviation
1	241	1	5	3.90	1.062
2	241	1	5	3.63	1.084
3	241	1	5	3.85	1.038
4	241	2	5	4.24	0.975
5	241	1	5	4.18	0.982
6	241	1	5	4.12	1.018
7	241	1	5	3.71	1.258
8	241	1	5	3.99	1.074
9	241	1	5	3.78	1.095
10	241	1	5	3.78	1.152
11	241	1	5	3.91	1.033
12	241	1	5	3.72	1.145
13	241	1	5	2.73	1.431
14	241	1	5	3.00	1.386
15	241	1	5	3.95	1.069
16	241	1	5	3.75	1.233
General	241	1.63	5	3.76	0.837

## DISCUSSION

During rehabilitation of a patient that needs urinary catheterization, nurses are very important to prepare the patient and/or health caregivers, in relation to capacitation and to management and purchasing of material. When patient and health-caregiver develop self-confidence for the procedure, performance is more efficient and motivates the rehabilitation process.

In this study, 241 subjects were interviewed with almost 50% of patients and 50% of health caregivers, both responsible for the rehabilitation of urinary bladder using clean intermittent urinary catheterization. The institution trained those individuals (where the study was performed) after primary diagnosis. The methods are perfected by initiatives of the university and the personal. Self-confidence is one of the several aspects that are been implemented (5).

Self-confidence may be related to the self-efficacy theory that is constantly associated to a behavior or task. It originates from repeated experiences and realistic perception of individual difficulties and potentials. Individuals with higher sense of self-confidence are prone to challenges and correction of failures (18, 19).

Self-confidence of clean intermittent urinary catheterization is related to success and continuity of treatment. Since there were no validated instruments to measure this aspect, it was proposed this question form (SCSCISC). In order to validate it, it was used an adequate number of participants according to observed results and the reference (16).

The proposed instrument showed high correlation of all items with total, indicating a correct reliability index (Alpha=0.944). SCSCISC measures self-confidence of patients and health caregivers.

Statistical and factorial analysis maintained all 16 items in one factor (56.5% of variance), corroborated by Screen-plot, showing adequacy of proposed items in the instrument.

Sample descriptive values related to self-confidence (Table-3) indicate that the higher values were related to hand hygiene and lower values related to “what to do when there is blood in the urine”.

Such results are in accordance to other studies and stress the easy incorporation of relevance related to hand hygiene, topic vastly debated for a long time by public services to prevent hospital infection by professionals (20). In relation to urethral trauma this is a difficulty also to professionals (21). The professional must recognize and use lubricants adequately, know potentials and difficulties of different compounds of urethral catheters and present the best evidences to patients and/or health caregivers during the learning process.

The limiting aspect of the present study is the use of sample of patients and health caregivers in one only set. However, when separately analyzed, the subsamples show the same results as the whole set. In view of the rehabilitation process of patients and the role of health caregivers, the authors decided to create a single instrument to measure self-confidence of urinary catheterization.

Self-confidence is intrinsically related to self-efficacy. Health professionals are responsible for the development, follow-up, motivation and measurement of self-confidence of patients and health caregivers in relation to intermittent urinary catheterization. Nurses are essentials for urinary rehabilitation and the proposed instrument is relevant in this process.

## CONCLUSIONS

This study validated a 16 items instrument (SCSCISC) in the form of a Likert scale to measure self-confidence of patients and health caregivers during intermittent urinary catheterization. It showed correct psychometric adequacy even when samples were singly studied (patients and health caregivers).

It is recommended replication of data and of the scale in new samples in order to evaluate self-confidence of patients and health caregivers during clean intermittent urinary catheterization, in order to improve quality of capacitation and treatment success.

## References

[1] Reis RB, Zequi SC, Zeratti M (2013). Urologia Moderna.

[2] Guyton AG, Hall JE (2011). Tratado de Fisiologia Médica.

[3] Sociedade Brasileira de Urologia (2008). Bexiga Urinária: Cateterismo intermitente. Projeto Diretrizes.

[4] Seth JH, Haslam C, Panicker JN (2014). Ensuring patient adherence to clean intermittent self-catheterization. Patient Prefer Adherence.

[5] Mazzo A, Souza VD, Jorge BM, Nassif A, Biaziolo CF, Cassini MF (2014). Intermittent urethral catheterization-descriptive study at a Brazilian service. Appl Nurs Res.

[6] Mangnall J (2013). Important considerations of intermittent catheterisation. NRC Journal.

[7] Newman DK, Willson MM (2011). Review of intermittent catheterization and current best practices. Urol Nurs.

[8] Bandura A (1993). Perceived self-efficacy in cognitive development and functioning. Educational psychologist.

[9] Bandura A (1995). Self-efficacy in changing societies.

[10] Gould CV, Umscheid CA, Agarwal RK, Kuntz G, Pegues DA, Healthcare Infection Control Practices Advisory Committee (2010). Guideline for prevention of catheter-associated urinary tract infections 2009. Infect Control Hosp Epidemiol.

[11] Geng V, Cobussen-Boekhorst H, Farrell J, Gea-Sánchez M, Pearce I, Schwennesen T (2012). Catheterisation: indwelling catheters in adults: urethral and suprapubic.

[12] Mazzo A, Gaspar AACS, Mendes IAC, Trevizan MA, Godoy S, Martins JCA (2012). Urinary catheter: myths and rituals present in preparation of patients. Acta Paul Enferm.

[13] Mazzo A, Martins JC, Jorge BM, Batista RC, Almeida RG, Henriques FM (2015). Validation of the self-confidence scale of nursing care in urinary retention. Rev Lat Am Enfermagem.

[14] Silva DR, Mazzo A, Jorge BM, Souza VD, Fumincelli L, Almeida RG (2015). Intermittent Urinary Catheterization: The Impact of Training on a Low-fidelity Simulator on the Self-confidence of Patients and Caregivers. Rehabil Nurs.

[15] Fumincelli L, Mazzo A, Jorge BM, Souza VD, Silva DRA (2014). Cateterismo urinário intermitente limpo: impacto do treino em simulador de baixa fidelidade na autoconfiança de pacientes e cuidadores.

[16] Pestana MH, Gageiro JN (2005). Análise de Dados para Ciências Sociais:A complementaridade do SPSS.

[17] Hair JF (2010). Multivariate Data Analysis.

[18] Bandura A (1983). Self-efficacy determinants of anticipated fears and calamities. J Personal Soc Psychol.

[19] Perry P (2011). Concept analysis: confidence/self-confidence. Nurs Forum.

[20] Brasil, Agência Nacional de Vigilância Sanitária (ANVISA) (2007). Higienização das mãos em serviços de saúde.

[21] Fink R, Gilmartin H, Richard A, Capezuti E, Boltz M, Wald H (2012). Indwelling urinary catheter management and catheter-associated urinary tract infection prevention practices in Nurses Improving Care for Healthsystem Elders hospitals. Am J Infect Control.

